# Diabetes and Aging: From Treatment Goals to Pharmacologic Therapy

**DOI:** 10.3389/fendo.2019.00045

**Published:** 2019-02-18

**Authors:** Miriam Longo, Giuseppe Bellastella, Maria Ida Maiorino, Juris J. Meier, Katherine Esposito, Dario Giugliano

**Affiliations:** ^1^Unit of Endocrinology and Metabolic Diseases, Department of Advanced Medical and Surgical Sciences, University of Campania Luigi Vanvitelli, Naples, Italy; ^2^Diabetes Division, St Josef Hospital, Ruhr-University Bochum, Bochum, Germany; ^3^Diabetes Unit, Department of Advanced Medical and Surgical Sciences, University of Campania Luigi Vanvitelli, Naples, Italy

**Keywords:** type 2 diabetes, elderly, diabetes-related comorbidities, glycemic targets, glucose lowering drugs

## Abstract

Diabetes is becoming one of the most widespread health burning problems in the elderly. Worldwide prevalence of diabetes among subjects over 65 years was 123 million in 2017, a number that is expected to double in 2045. Old patients with diabetes have a higher risk of common geriatric syndromes, including frailty, cognitive impairment and dementia, urinary incontinence, traumatic falls and fractures, disability, side effects of polypharmacy, which have an important impact on quality of life and may interfere with anti-diabetic treatment. Because of all these factors, clinical management of type 2 diabetes in elderly patients currently represents a real challenge for the physician. Actually, the optimal glycemic target to achieve for elderly diabetic patients is still a matter of debate. The American Diabetes Association suggests a HbA1c goal <7.5% for older adults with intact cognitive and functional status, whereas, the American Association of Clinical Endocrinologists (AACE) recommends HbA1c levels of 6.5% or lower as long as it can be achieved safely, with a less stringent target (>6.5%) for patients with concurrent serious illness and at high risk of hypoglycemia. By contrast, the American College of Physicians (ACP) suggests more conservative goals (HbA1c levels between 7 and 8%) for most older patients, and a less intense pharmacotherapy, when HbA1C levels are ≤6.5%. Management of glycemic goals and antihyperglycemic treatment has to be individualized in accordance to medical history and comorbidities, giving preference to drugs that are associated with low risk of hypoglycemia. Antihyperglycemic agents considered safe and effective for type 2 diabetic older patients include: metformin (the first-line agent), pioglitazone, dipeptidyl peptidase 4 inhibitors, glucagon-like peptide 1 receptor agonists. Insulin secretagogue agents have to be used with caution because of their significant hypoglycemic risk; if used, short-acting sulfonylureas, as gliclazide, or glinides as repaglinide, should be preferred. When using complex insulin regimen in old people with diabetes, attention should be paid for the risk of hypoglycemia. In this paper we aim to review and discuss the best glycemic targets as well as the best treatment choices for older people with type 2 diabetes based on current international guidelines.

## Introduction

Life expectancy is defined as the average number of years that a newborn is expected to live assuming that current mortality rates remain the same throughout its life. Global average life expectancy has increased by 5.5 years between 2000 and 2016, with the fastest increase since the 1960s, as a consequence of declining number of deaths from infectious causes (1). Latest estimates of life expectancy at birth were of 80.9 years across the 28 European member states (2) and 78.9 years in United States of America (USA) (3). The progressive decline of age-standardized rates of death from non-communicable chronic diseases (NCDs, cardiovascular and respiratory diseases, cancer, and diabetes) registered globally between 2006 and 2016 (4), together with the rising number of people older than 65 years, especially in westernized countries, has led to an increased prevalence of NCDs among elderly, resulting in more years of life spent with morbidity and disability (5).

Diabetes is recognized as an important cause of premature death and disability. In the past three decades the age-standardized prevalence of diabetes has risen substantially in countries at all income levels; 40% of this increase is estimated to result from population growth and aging (6). Therefore, diabetes is one of the most widespread health burning problems in the elderly, which represent a heterogeneous and complex population as it include both newly diagnosed older diabetic patients and patients with long-standing diabetes with onset in middle or early age (7). Consequently, management of diabetes in elderly subjects is particularly complex and challenging for clinicians, due to difficulty in individualizing glycemic targets, treatment strategies, coexisting comorbidities, polypharmacy, and hypoglycemic risk. The aim of this review is to discuss the best glycemic targets as well as the best treatment choices for old people with type 2 diabetes based on current shared international guidelines.

## Epidemiology

Type 2 diabetes represents the most common metabolic disease in older adults. According to the latest estimates of the International Diabetes Federation (IDF), diabetes shows a high prevalence in people older than 65 years (8). In 2017, the number of diabetic people aged 65–99 was estimated to be 122.8 million (around 18% of prevalence rate), of whom 98 million had <80 years (65–79 years); these numbers are expected to easily exceed 200 million in 2045 (8). China, United States of America and India are the countries with highest numbers of people older than 65 with diabetes. Similar prevalence rates of diabetes were found in the European Region, reaching values ranging between 14.9 and 25.0% (8). The main reasons imputable to this spreading may be found in the longer life expectancy, the global diffusion of both unhealthy lifestyle habits and environmental pollution (9).

The number of deaths caused by diabetes in the age range of 60–99 years in 2017 was 3,200,000, which represents ~60% of deaths due to diabetes among the age group between 18 and 99 (8). Moreover, elderly diabetic patients are exposed to a higher risk of cardiovascular complications, including peripheral vascular disease, heart disease, and stroke (10), and many geriatric syndromes (from cognitive impairment to urinary incontinence) (11).

## Pathophysiology of Diabetes in Elderly

Several factors participate in the pathophysiology of diabetes in older age. Chronological age per sè represents a risk factor for many chronic diseases (12). Advanced age leads to the exacerbation of systemic chronic inflammation, oxidative stress, DNA damage, decline of mitochondrial function, cellular senescence, and tissue dysfunction, all conditions which contribute to generate metabolic disorders (13). Indeed, aging is associated with raised levels of pro-inflammatory molecules, including interleukin (IL) 1, IL-6, IL-8, IL-13, IL-18, C-reactive protein, interferons α and β, transforming growth factor β (TGF-β), tumor necrosis factor α (TNF-α), and serum amyloid (14). Furthermore, the age-related variation of body composition leads to an increase in fat mass, especially visceral adiposity, and an equal decrease in lean and skeletal mass (15). With aging, there is a decline in preadipocyte replication and an expansion of senescent cells in adipose tissue which enhance lipotoxicity and favor the generation of a pro-inflammatory status (16). Moreover, some studies have showed that aging (1) impairs insulin secretion from β-cells in response to endogenous incretins (GIP), (2) is associated with reduced insulin sensitivity, and (3) promotes β-cell death by inducing mitochondrial dysfunction (14). In older subjects, abnormalities in both insulin sensitivity and insulin secretion lead gradually to impaired glucose tolerance and consequently to clinically manifest diabetes. Postprandial hyperglycemia is a characteristic feature of type 2 diabetes in older patients. Therefore, an oral glucose tolerance test should be performed in older subjects with impaired fasting glucose to early detect diabetes, which otherwise could be undiagnosed using fasting plasma glucose alone (7).

## Diabetes and Geriatric Syndromes

**Figure 1:**
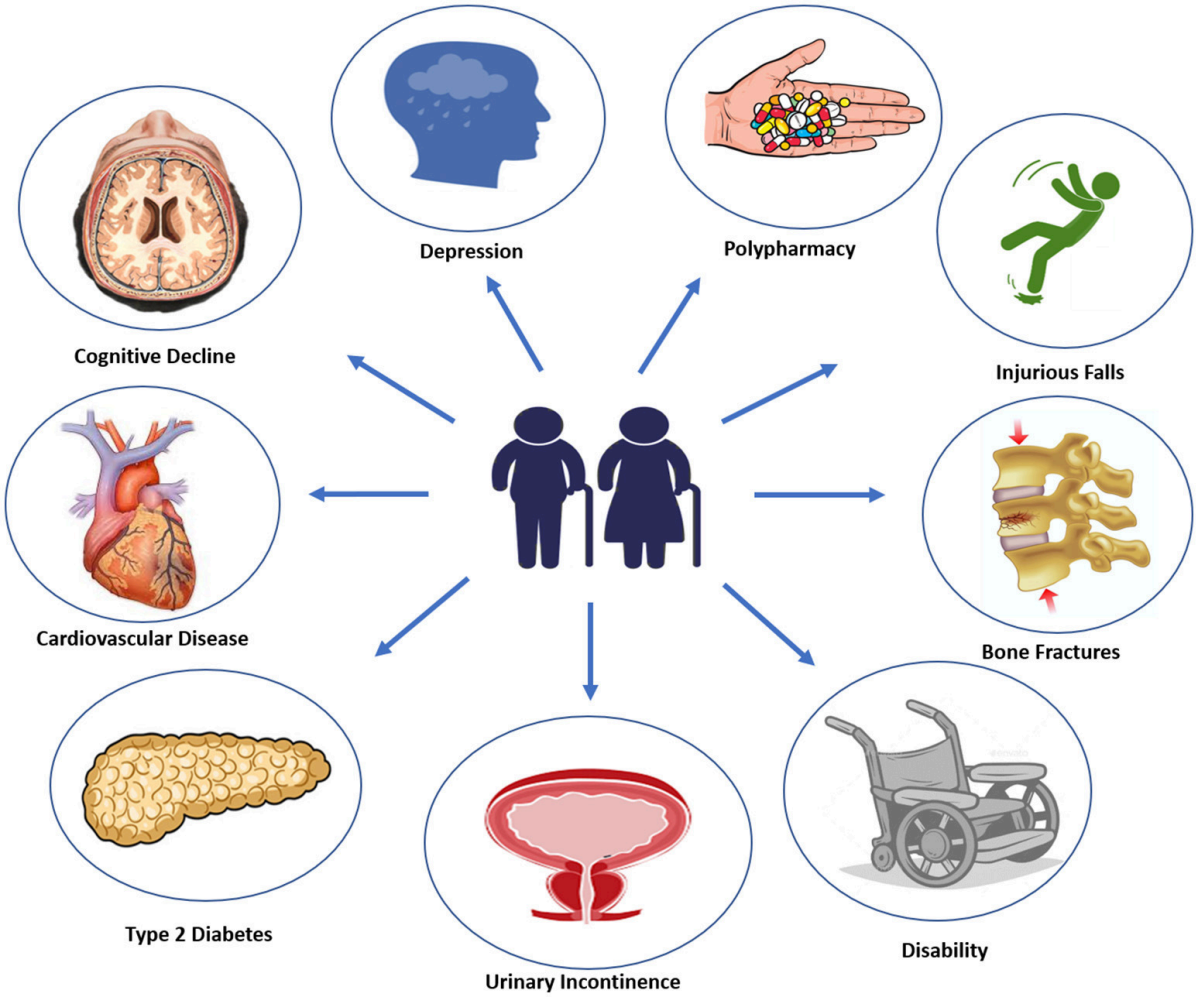
Most common clinical conditions associated with aging.

Diabetes onset in elderly usually manifest with vague and not specific symptoms, such as dehydration, dry mouth, confusion, fatigue, lethargy, weight loss, and an increased tendency toward genitourinary infections (17). It has been estimated that 60% of older patients with type 2 diabetes has at least one other comorbid disease, and 40% of these patients has actually no <4 concurrent illnesses (18). Most common type 2 diabetes comorbidities, including cognitive impairment, disability, depression, apathy, urinary incontinence, polypharmacy, hearing, and visual impairment, falls and fractures, fall under geriatric syndromes (19) (Figure 1). With advanced age, malnutrition, physical inactivity, and unwanted weight loss become more frequent. Moreover, elderly diabetic patients are more likely to experience severe or unaware hyper/hypoglycemic episodes and major adverse cardiovascular events (MACE), due to peripheral and autonomic neuropathy. Therefore, a comprehensive geriatric assessment including screening for microvascular complications, cardiovascular risk factors, and geriatric syndromes should be performed at initial diagnosis of diabetes in elderly patients (20).

### Cognitive Dysfunction and Depression

There is evidence that type 2 diabetes is associated with cognitive dysfunctions. Older diabetic patients have higher risk to develop mild cognitive impairment (MCI), all-cause dementia and Alzheimer's disease (21). Specific mechanisms underlying this association are still unclear; however, main factors involved are vascular dysfunction, high blood pressure, hyperglycemia, hypoglycemic events, insulin resistance, and neuroinflammation (22). Furthermore, depressive and apathic symptoms frequently co-exist with diabetes (23), and some studies have found that combination of diabetes and depression may express a toxic effect on the brain, increasing the risk for dementia (24). In light of this, the American Diabetes Association (ADA) recommends for subjects over 65 years old (with a level of evidence B) a neuro-psychological screening at the initial visit and annually to early detect mild cognitive impairment and depression, by using some specific test (Mini-Mental State Examination, Montreal Cognitive Assessment and Geriatric Depression Scale), and minimizing hypoglycemic events to reduce the risk of MCI (25).

### Disability, Fractures and Urinary Incontinence

Type 2 diabetes in elderly is a powerful risk factor for functional limitations, frailty, loss of independence, and disability (26). Moreover, there is evidence that type 2 diabetes increases the risk of fracture risk and secondary hypogonadism, which also contribute to enhance risk of osteoporosis and muscle weakness in men (27, 28). With aging there is a progressive loss of strength and toughness of skeletal and muscle mass which leads to a status of osteo- and sarcopenia. Changes in skeletal muscle protein turnover could accelerate these alterations in type 2 diabetic patients (29), resulting in a greater risk of falling and bone fractures (30). As testosterone decline with advancing age, the assessment of its concentrations may be useful in case of signs and symptoms of overt hypogonadism to better evaluate the risk of fracture in this selected population (31, 32). Indeed, there is evidence that older patients with type 2 diabetes have an increased risk of hip fractures, particularly in insulin-treated patients, and non-skeletal fall injuries (33). A moderate but regular physical activity and a high adherence to Mediterranean dietary pattern showed some benefits in reducing the risk of falls and physical impairments in patients older than 75 years (34, 35). The American Geriatrics Society suggests to interrogate older patients about falls at least every 12 months, examine potentially reversible causes of falls (medications, environmental factors, limiting factors) and perform a complete basic evaluation when an injurious fall occurs (level of evidence III, strength B) (36).

Urinary incontinence is a frequent comorbidity of diabetes, although it is usually not-reported by patients (37). Therefore, according to the American Geriatrics Society, physicians should always perform an annual screening for urinary incontinence which may be an important cause of social isolation, depression, falls, and fractures (level of evidence III, strength A) (36).

### Overtreatment and Polypharmacy

Both overtreatment and polypharmacy are very common among frail older diabetic subjects. The prevalence of polypharmacy regimen, defined as the use of more than 5 medications, increases with age. Results from a Dutch study revealed that 64 persons (20%) out of 319 type 2 diabetic patients aged ≥70 years were overtreated and frail (38). Furthermore, one-quarter of US older diabetic adults are on potential overtreatment for tight glycemic control using glucose-lowering medications at high risk of hypoglycemia (39). In a cohort of 8,932 adults with diabetes, 78% of patients had polypharmacy, which was more likely associated with age ≥60 years, female sex, and coexisting chronic diseases (40). Polypharmacy in older diabetic patients may produce detrimental effects mainly due to increased risk of drug-drug interactions and adverse side effects (41). However, a deintensification rather than intensification of pharmacological therapy should be advisable in diabetic patients in older age, in consideration of both benefits and risks associated with complex therapeutic regimens. Moreover, older adults with diabetes should annually update the list of used medications for their own clinicians (level of evidence II, strength A) (36).

## Glycemic Control

Older patients represent a very heterogeneous and challenging population concerning diabetes care and treatment. While treating diabetes in elderly, clinicians should be always aware of maintaining a good quality of life. Patient-centered glycemic targets are needed in order to achieve the glycemic control avoiding dangerous or extreme glucose excursions. Elderly patients are highly vulnerable to hypoglycemic events, as a consequence of progressive age-related decrease in β-adrenergic receptor function. Indeed, hypoglycemia in older age has been associated with an increased risk to develop cognitive impairment, dementia, all-cause hospitalization, and all cause mortality (42–44). Use of insulin or insulin secretagogues, polypharmacy, coexisting comorbidities, renal insufficiency, dehydration, impairment of counter-regulatory responses represent the main predisposing risk factors for hypoglycemic episodes (45). Assessment of potential risk factors for hypoglycemia is an important part of the clinical management of older diabetic subjects. Moreover, both patients and caregivers have to be trained and well-educated on the prevention, detection, and treatment of hypoglycemic events (11). On the other hand, both untreated or undertreated hyperglycemic events should be avoided in old people, given the higher risk of dehydration, dizziness, falls, and long-term mortality (46).

The paucity of randomized controlled trials (RCTs) for diabetes treatment in older adults does not allow to clearly establish the most appropriate therapeutic goals in the elderly. Three major high-profile trials (ACCORD, VADT, and ADVANCE trials) (47–49) conducted on type 2 diabetic people aged around 60 years old showed that achieving tight glycemic control (HbA1c < 6% or < 6.5%) was not associated with improvements in cardiovascular outcomes, and one of them (47) has been stopped earlier because of increased mortality in the intensive glucose control arm (number of death in intensive vs. standard therapy, 257 vs. 203, HR 1.22; *P* = 0.04) and increased hypoglycemic events (538 vs. 179, *P* < 0.001). On the other hand, a large observational study reported that an HbA1c level > 8% was associated with increased risk of all-cause, cardiovascular, and cancer mortality in older adults with diabetes (50). Actually, the best glycemic target to achieve for elderly diabetic patients is still a matter of debate (51). However, there is agreement on tailoring glycemic goals in function of patient's life expectancy, diabetes duration, functional status, existing comorbidities, and pursuing moderate (HbA1c between 7 and 8%) rather than tight control (52) in old diabetic patients.

## What Do Current International Guidelines Say on Glycemic Goals?

Table 1 summarizes the glycemic goals for elderly affected by diabetes according different international guidelines. The current Standards of Medical Care in Diabetes 2019 released by American Diabetes Association (ADA) indicate an HbA1c goal < 7.5% for healthy older adults with intact cognitive and functional status and a fasting or pre-prandial glucose between 90 and 130 mg/dL, whereas less stringent targets (HbA1c < 8.0–8.5%) may be advisable for frail older adults with limited life expectancy, with fasting glucose level between 100 and 180 mg/dL (25). These therapeutic objectives are in line with those for adults older than 65 years indicated by American Geriatrics Society (HbA1c ranging between 7.5 and 8%), which suggest to determine HbA1c at least every 6 months, or more frequently if needed (36). Beyond tailored glycemic goals, ADA highlights the importance of controlling any other cardiovascular risk factor with an appropriate lipid-lowering, anti-platelet, and anti-hypertensive therapy.

**Table 1:** Glycemic targets in elderly patients according to the current international guidelines.

**International Guidelines, year**	**HbA1c goal for most healthy older adults with intact cognitive and functional status**	**HbA1c goal for most frail older adults, with multiple comorbidities and limited life expectancy**
ADA, 2019	<7.5%	< 8–8.5%
AGA, 2013	7–7.5%	7.5–9%
AACE, 2018	≤ 6.5%	>6.5%
ACP, 2018	7–8%	No specific target but minimizing symptoms related to hyperglycemia

*ADA, American Diabetes Association; AGA, American Geriatrics Association; AACE, American Association of Clinical Endocrinologists; ACP, American College of Physician*.

Differing from ADA, the American Association of Clinical Endocrinologists (AACE) advises an HbA1c goal of 6.5% or lower for most patients without history of cardiovascular diseases (CVD) as it can be safely achieved, whereas, a broader HbA1c target (>6.5%) is suggested for older patients with concurrent serious illness, high risk of hypoglycemia, and limited life expectancy, as the patient does not experience characteristic hyperglycemic symptoms (polydipsia, polyuria, polyphagia) (53).

On the other hand, the American College of Physicians (ACP) suggests more conservative goals (HbA1c levels between 7 and 8%) for most older patients, and a less intense pharmacotherapy when HbA1c ≤ 6.5% (54). Moreover, for patients over 80 years old and with important serious chronic diseases (dementia, cancer, end-stage kidney disease, respiratory, and heart disease) clinicians should focus on minimizing symptoms related to hyperglycemia and avoiding an HbA1c target in patients with a life expectancy <10 years (54). Despite discrepancies in international guidelines (55), the mantra that every physician should follow could be resumed in “treat the patient, not the HbA1c level” (56).

## Diabetes Treatments

Studies comparing the effectiveness of anti-diabetes drugs in elderly are lacking, due to the exclusion of older diabetic adults from RCTs, given the high number of comorbidity and their enhanced cardiovascular risk. Every therapeutic strategy should be chosen considering age, health status, self-manageability, cognitive and nutritional status, and comorbidities (Table 2). Generally, in older adults at higher risk to experience hypoglycemic events, medications with low risk of hypoglycemia should be preferred. Furthermore, it is advisable to simplify poly-pharmacological regimens in order to reduce adverse effects and achieve most appropriate glycemic goals. The latest consensus on the management of hyperglycemia in type 2 diabetes of the ADA and the European Association for the Study of Diabetes (EASD) (57) recommends to use drugs with proven cardiovascular benefit in patients with established clinical cardiovascular disease. Anti-hyperglycemic agents considered safe and effective for type 2 diabetic older patients can be divided in oral and injectable drugs (Table 3).

**Table 2:** Most frequent clinical phenotypes in elderly with suggested HbA1c target and glucose-lowering treatment.

**Phenotype**	**Comorbidities**	**Diabetic complications**	**Glycemic target**	**Glucose-lowering treatment**
75-year old men HbA1c 7.2% Treated with metformin 1,500 mg/day	Hypertension	None	HbA1c <7.0%	Consider to titrate metformin or add a DPP-4 inhibitor
78-year old woman HbA1c 7.6% Treated with metformin 2000 mg/day	Heart failure (NYHA class III) Osteoporosis CKD (GFR 48)^*^	Peripheral neuropathy	HbA1c <7.5%	Suspend metformin Consider to start a SGLT2-inhibitor and in second instance a GLP-1RAs or a DPP-4 inhibitor
81-year old men HbA1c 8.4% Treated with Glargine U/day 26	Cerebrovascular disease MCI CKD (GFR 38)^*^ Prostate adenoma	Diabetic ulcer of the right foot	HbA1c <8.0%	Consider to add a GLP-1 RAs (liraglutide, lixisenatide or dulaglutide) or a DPP-4 inhibitor, or to switch to a fixed ratio combo of basal insulin and GLP-1RA
80-year old woman HbA1c 8.7% Treated with a combo of metformin and sulphonilurea 800 + 5 mg/day	Metastatic breast cancer CKD (GFR 29)^*^ Coronary heart disease Recurrent symptomatic hypoglycemia Wasting syndrome	Autonomic neuropathy	HbA1c <8.5%	Suspend metformin and sulphonilurea. On the basis of SBGM, consider to start pioglitazone or a DPP-4 inhibitor or a basal insulin

* *GFR is estimated as mL/min/1.73 m^2^ of body surface*.

*CKD, chronic kidney disease; DPP-4, dipeptidyl peptidase-4; GFR, glomerular filtration rate; GLP-1 RAs, glucagon-like peptide-1 receptor agonists; MCI, mild cognitive impairment; SBGM, self blood glucose monitoring; SGLT2, sodium-glucose co-transporter 2*.

**Table 3:** Glucose-lowering medications available in Europe with specific characteristics to drive the treatment choice for old people with type 2 diabetes.

**Anti-hyperglycemic class**	**Mechanism of action**	**General characteristics**	**Potential side effects**	**Contraindications**
Biguanides *Metformin*	Insulin sensitizer agent, lowering glucose concentration by reducing hepatic gluconeogenesis	First line agent in type 2 diabetes. Good efficacy, low cost, no risk of hypoglycemia	Gastrointestinal symptoms, rare lactic acidosis	GFR^*^ <30 Dose reduction if GFR 30–45
Thiazolidinediones *Pioglitazone*	Insulin sensitizer agent, influencing transcriptional processes by activation of PPAR-γ	Good efficacy, low cost, no risk of hypoglycemia	Weight gain, fluid retention, increased risk of bone fracture and bladder cancer	CHF (NYHA class III-IV), DKA
Sulfonylureas *Glibenclamide* *Glicazide* *Glimepiride* *Glipizide*	Insulin secretagogue agents, acting on SUR subunit of ATP-sensitive K+ channels in pancreatic beta cells	High efficacy, low cost. Short-acting ones preferred in older patients	Hypoglycemia, weight gain	Severe kidney or liver disease. Long-acting ones should not be used in elderly
Meglitinides: *Netaglinide* *Repaglinide*	Insulin secretagogue agents, enhancing early phase of insulin secretion	High efficacy in lowering postprandial glucose levels, low cost. Safe in advanced renal disease with dose adjustment	Hypoglycemia, weight gain	DKA, adrenal insufficiency, hypopytuitarism
DPP-4 inhibitors *Alogliptin* *Linagliptin* *Sitagliptin* *Saxagliptin* *Vildagliptin*	Incretin enhancer agents, they inhibit the DPP-4 enzyme extending GLP-1 life-time, leading to increased insulin secretion and decreased glucagon secretion in a glucose dependent manner	Intermediate efficacy, neutral effect on weight, well-tolerated, no risk of hypoglycemia in monotherapy, proven cardiovascular safety, intermediate cost	Potential risk of pancreatitis. Saxagliptin is associated with higher risk of heart failure hospitalization	Previous episode or risk of pancreatitis. Dose adjustment in moderate to severe kidney disease except for linagliptin. Saxagliptin is contraindicated if GFR^*^ <15
SGLT2 inhibitors *Canagliflozin* *Dapagliflozin* *Empagliflozin*	Glycosuric agents, they inhibit the Na/Glucose renal cotransporter on kidney proximal convoluted tubule, increasing urinary glucose concentration, and favoring osmotic diuresis	High efficacy, reduced body weight and blood pressure, no risk of hypoglycemia, benefit on cardiovascular and renal outcomes, high cost	Mycotic genital infections, de-hydration, orthostatic hypotension, increased risk of DKA, lower extremities amputations (canaglifozin), bone fracture	GFR^*^ ≤ 30. If used with diuretics dose adjustment is needed
GLP-1RAs short-acting *Exenatide* *Lixisenatide* GLP-1RAs long-acting *Albiglutide* *Dulaglutide* *Exenatide LAR* *Liraglutide* *Semaglutide*	Incretin analogs, activating GLP-1 receptors, thus promoting insulin secretion and decreasing glucagon secretion in a glucose dependent manner, slowing gastric emptying and favoring sense of satiety	High efficacy, no risk of hypoglycemia, weight loss, once-daily or once weekly injection, benefit on cardiovascular outcomes (liraglutide, semaglutide, and albiglutide), high cost	Nausea, vomiting, diarrhea, modestly increase heart rate, potential risk of pancreatitis and thyroid cancer, gallbladder stones	Previous episode or risk of pancreatitis, thyroid cancer, multiple endocrine neoplasia syndrome type 2 (MEN 2), severe kidney disease or dialysis (liraglutide and dulaglutide can be used until GFR^*^> 15)
Long acting insulin analog *Degludec* *Detemir* *Glargine*	Basal recombinant insulin analogs activating insulin receptor, lowering glucose levels	Very high efficacy, once-daily injection, frequent dose adjustment for optimal efficacy, high cost	Weight gain, hypoglycemia, lipoatrophy, injection site reaction	Hypersensitivity to insulin or its excipients
Short acting insulin analog *Aspart* *Glulisine* *Lispro*	Pre-meal recombinant insulin analogs activating insulin receptor, lowering glucose levels	Very high efficacy, high risk of hypoglycemia, multiple daily frequent dose adjustment for optimal efficacy, high cost	Weight gain, hypoglycemia, lipoatrophy, injection site reaction	Hypersensitivity to insulin or its excipients
Ultra rapid acting insulin analog *Faster aspart*	Pre-meal recombinant insulin analogs activating insulin receptor, lowering glucose levels	Very high efficacy, high risk of hypoglycemia, multiple daily frequent dose adjustment for optimal efficacy, high cost	Weight gain, hypoglycemia, lipoatrophy, injection site reaction	Hypersensitivity to insulin or its excipients

* *GFR is estimated as mL/min/1.73 m^2^ of body surface*.

*CHF, chronic heart failure; DKA, diabetic ketoacidosis; DPP-4, dipeptidyl peptidase-4; Exenatide LAR, exenatide long acting release; GFR, glomerular filtration rate; GLP-1 RAs, glucagon-like peptide-1 receptor agonists; PPAR-γ, peroxisome proliferating activated receptor-γ; SGLT2, Sodium-glucose co-transporter 2; SUR, sulfonylurea receptor*.

### Oral Anti-hyperglycemic Drugs

Metformin is the first-line medication recommended in the management of type 2 diabetes. It reduces both insulin-resistance and hepatic gluconeogenesis, lowering glucose concentrations without increasing hypoglycemic risk. The starting dose is of 500 mg once or twice a day to be assumed with meals up to 2,500 mg/day at the maximum dose. Moreover, a once daily extended-release formulation of metformin is now available, which is associated with a better gastrointestinal tolerability profile and patients' compliance. As it is excreted by the urine, a good glomerular filtration rate is needed (58). Therefore, a dose reduction has to be considered if glomerular filtration rate (GFR) is between 30 and 45 mL/min/1.73 m^2^, while discontinuation is recommended if GFR < 30 mL/min/1.73 m^2^ (59). The main adverse effects described are commonly gastrointestinal symptoms and very rarely lactic acidosis. It is a safe and effective anti-hyperglycemic drug, with low cost, and minimal risk of hypoglycemia. Nevertheless, it should be carefully used under conditions of congestive heart failure and hepatic dysfunction, which could increase the risk of lactic acidosis (25).

Thiazolidinediones also act as insulin sensing agent influencing transcriptional processes by activation of peroxisome proliferator-activated receptor-γ (PPAR-γ). Pioglitazone is the only one remaining drug of this class, as it has proven to be safe in the presence of cardiovascular disease (60). It is characterized by good efficacy, low cost, and no risk of hypoglycemia when used in monotherapy. It can be used even in case of low GFR value (61) starting from the lowest dose of 15 mg to the maximum dose of 45 mg with meals. Pioglitazone is associated with weight gain and fluid retention, so that it is contraindicated in case of congestive heart failure (NYHA class III, IV). Furthermore, it is not advisable to use the drug in older person at risk for falls because it has proven to increase risk of non-osteoporotic bone fractures (62). Finally, it is contraindicated in patients with or at high risk for bladder cancer (63).

Sulfonylureas are an insulin secretagogue class, which act by favoring β-cells membrane depolarization and consequently insulin secretion. They are characterized by high glucose lowering efficacy and low cost, but they should be used with extreme caution because of the high risk of hypoglycemia and weigh gain. Short acting ones with lowest hypoglycemic risk, such as gliclazide, should be preferred in older diabetic patients, when initial therapy with metformin is contraindicated or not tolerated (64). By contrast, long acting sulfonylureas, as glibenclamide, are considered inappropriate in elderly diabetes management.

Metiglinides are short-acting insulin secretagogue agents, that enhance early phase of insulin secretion at meals, lowering postprandial glucose levels. They present lower risk of hypoglycemia than sulfonylureas, since their activity is dependent on the presence of glucose (20). Repaglinide is the most effective agent of this class, with a moderate effect on weight gain. Use of repaglinide may be indicated for elderly patients with type 2 diabetes because of the low risk of hypoglycemia, high efficacy on postprandial hyperglycemia, and safe use in renal impairment (65).

Dipeptidyl peptidase 4 (DPP-4) inhibitors belong to the class of incretin enhancer agents. They inhibit the DPP-4 enzyme, thereby extending the life-time of GLP-1 and increasing insulin secretion in a glucose dependent manner. Drugs in this class are generally well-tolerated in older people, with neutral effect on body weight and very low risk of hypoglycemia (66, 67). DPP-4 inhibitors have proven to be effective in reducing baseline HbA1c levels and fasting plasma glucose (68). Moreover, a study of 80 elderly diabetic patients treated with oral glucose-lowering drug (DPP4-inhibitors or sulfonylureas) for at least 24 months showed that patients using DPP-4 inhibitors had better sarcopenic parameters (fat-free mass, skeletal muscle mass, and related indices, muscle strength, and gait speed) as compared with those receiving sulfonylureas (69). The cardiovascular safety of this class of agents has been confirmed by several randomized controlled trials (70–74). Alogliptin, saxagliptin, sitagliptin, and linagliptin (70–74) have proven to neither increase nor decrease risk of the combined major adverse cardiovascular events (MACE) in type 2 diabetic patients with established cardiovascular disease. However, in the SAVOR-TIMI 53 study (72), saxagliptin, showed a 27% increased risk of hospitalization for heart failure (HF) among patients with elevated levels of natriuretic peptides, previous heart failure, or chronic kidney disease, as compared with placebo (75). In the EXAMINE trial, patients with type 2 diabetes and recent acute coronary syndromes assigned to alogliptin had an increased, although non-statistically significant, rate of HF hospitalization when compared to the placebo group (76). Recently, in the TECOS trial, sitagliptin showed neutral effects on cardiovascular risk without any significant risk of HF hospitalization when compared with placebo in patients aged ≥75 years with well-controlled type 2 diabetes and cardiovascular disease (77). Moreover, data from the TECOS trial report that sitagliptin is not associated with a higher fracture risk, major osteoporotic fractures, or hip fractures (78). Therefore, DPP-4 inhibitors may be considered as an effective and safely treatment option for older patients with type 2 diabetes (79).

Sodium-glucose cotransporter 2 (SGLT-2) inhibitors are the latest marketed oral anti-hyperglycemic agents in diabetes management. These molecules act with an innovative and different mechanism of action: they inhibit Na/glucose renal cotransporter on kidney proximal convoluted tubule, increasing urinary glucose concentration, and favoring osmotic diuresis (diuretic effect). Beyond glucose lowering efficacy, SGLT-2 inhibitors have also beneficial effects in reducing body weight and blood pressure. Their use is permitted until GFR ≥ 30 mL/min/1.73 m^2^, due to safety concerns and lack of dedicated study in diabetic population with severe chronic renal disease. If SGLT-2 inhibitors are used in combination with diuretics, lowering the dose of diuretics is needed to minimize the risks of hypotension and dehydration (79). SGLT2-inhibitors are generally well-tolerated in older adults, except for increased risk of mycotic genital infections in both sexes. There is evidence from cardiovascular outcome trials (80, 81) that this class has beneficial effects in reducing the composite endpoint of cardiovascular deaths, non-fatal myocardial infarction and non-fatal stroke as compared with placebo in patients with type 2 diabetes and high cardiovascular risk. Similarly, in the multinational, observational CVD-REAL study, new users of empaglifozin, canaglifozin, and dapaglifozin reported lower risk of cardiovascular mortality, MACE and hospitalization for heart failure as compared with new users of other glucose-lowering drugs (82). Moreover, a subgroup analysis of the EMPA-REG OUTCOME study showed a significant reduction in the risk of MACE especially in patients older than 65 years treated with empaglifozin (80). Based on these results, ADA and EASD recommend their use in patients with established or at high risk of cardiovascular disease (57). In the respective RCTs designed to test the efficacy and safety of SGLT-2 inhibitors on renal outcomes (83, 84), both empagliflozin and canagliflozin use was associated with reduced risk of sustained loss of kidney function, attenuated GFR decline, and a reduction in albuminuria, which supports a possible renoprotective effect of this drugs in people with type 2 diabetes. More recently, treatment with dapagliflozin, compared with placebo, produced a significant 24% risk reduction in renal composite events, namely ≥40% decrease in eGFR below 60 ml/min/1.73 m^2^ of body-surface area, new end-stage renal disease, or death from renal or cardiovascular causes (85). Conversely, on May 2015 the Food and Drug Administration released a warning relative to an increased risk of diabetic ketoacidosis (DKA) associated with use of SGLT-2 inhibitors (86), on the basis of a comparative evaluation with DPP-4 inhibitors on a cohort of more than 140,000 type 2 diabetic patients (87). The increased incidence of DKA related to SGLT2-inhibitors may be probably related to the non-insulin-dependent glucose clearance, hyperglucagonemia, and volume depletion (88). Therefore, although this class has many beneficial effects on cardiovascular and renal outcomes, caution is needed using SGLT2 inhibitors in elderly because of increased risk of genital infections, dehydration, orthostatic hypotension, lower extremities amputations, and bone fracture (89, 90).

### Injectable Anti-hyperglycemic Drugs

Glucagon-like peptide 1 receptor agonists (GLP-1RAs) are innovative and pleiotropic drugs that act by promoting insulin secretion and reducing glucagon secretion in a glucose dependent manner and favoring weight loss. As they use the injectable way of administration, they require neuro-psychological and physical integrity. GLP-1RAs are highly effective in lowering glucose levels, with minimal risk of hypoglycemia (91, 92). Recently, a phase III RCT showed the superiority of lixisenatide as compared with placebo in reducing HbA1c levels and postprandial hyperglycemia in patients ≥70 years uncontrolled on their current antidiabetic treatment (93). The main adverse effects associated with GLP-1RAs use consist of nausea, vomiting, diarrhea, and an increase in heart rate (94). Furthermore, there is strong evidence from RCTs (95–97) that these drugs can reduce the risk of MACE in type 2 diabetic patients with high cardiovascular risk. Results from preclinical studies showed also favorable effects of GLP-1RAs on neuronal protection and cognitive performances (98, 99). Randomized controlled trials assessing effects of incretin therapy on cognitive function and Alzheimer's disease in humans are currently ongoing. If these benefits will be confirmed, use of GLP-1RA may be a helpful option even in patients with mild cognitive impairment.

Free and fixed-ratio combinations of GLP-1RAs and basal insulin formulations have been approved by regulatory agencies to potentiate antihyperglycemic effects and glycemic control in type 2 diabetic patients (57, 100). At the moment, two fixed-ratio combinations, insulin glargine plus lixisenatide (IGlarLixi) and insulin degludec plus liraglutide (IDegLira), have been approved for treatment of type 2 diabetes (101). A recent analysis compared effectiveness of fixed-ratio combination iGlarlixi vs. sequential administration of iGlar + Lixi in glucose control in type 2 diabetic patients (102). IGlarLixi was associated with significantly higher HbA1c reductions, weight loss and number of patients reaching HbA1c target despite lower insulin doses, with similar rates oh hypoglycemic events and lower rates of gastrointestinal adverse events. A meta-analysis of 26 RCTs have shown a mean reduction of 0.47% in HbA1c level associated with a mean weight loss of 2.5 Kg favoring the insulin/GLP-1RA combination as compared with other injectable anti-diabetes treatments, with no increased risk of hypoglycaemia (103). Moreover, when compared with intensive insulin therapy, either free or fixed combination of GLP-1RA and basal insulin led to a greater mean decrease of 0.53% in HbA1c level, a higher proportion of patients at HbA1c target of < 7% and reduction in body weight (104). Based on this evidence, combination strategies, either free or fixed, represent a good option for intensifying basal insulin therapy in patients with type 2 diabetes who need amelioration of glycemic control, without increasing the risk of hypoglycemia and weight gain (104).

Insulin remains the most effective drug for type 2 diabetes (105). The main limitations of insulin therapy are the risk of hypoglycemia and weight gain, although it can be administered at any GFR value. Insulin therapy requires patients' autonomy, intact visual, motor, and cognitive ability in diabetes management (25). Since its discovery in 1921, several and innovative insulin formulations have been developed. Insulin glargine (U100 or U300), degludec (U100 or U200), and detemir represent long acting insulin analogs which provide daily basal insulin profiles (106). A recent meta-analysis reported that insulin glargine U300 was as effective as glargine U100 in type 2 diabetic patients aged >65 years, with a reduced risk of nocturnal hypoglycemia (107). Compared with human insulin neutral protamine Hagedorn (NPH), long-acting insulin analogs have a longer duration of action and a fatter pharmacokinetic profile, with a reduced risk of hypoglycemia (106). Therefore, the newer basal insulins should be preferentially used in diabetic elderly, where they may be indicated as starting insulin therapy. Prandial rapid (aspart, lispro, glulisine) and ultra-rapid acting (faster aspart) insulin analogs used at mealtime can be combined with basal insulin to sooner improve and intensify glycemic control (108). However, both basal and prandial insulin require frequent titration to achieve the best anti-hyperglycemic effects. Patients on enteral or parenteral nutrition may require frequent glucose monitoring (intervals of 4–6 h) to better titrate the insulin dose and to avoid hypo- and hyperglycemic events (64). Caution is needed in insulin titration because a simple error can easily precipitate major hypoglycemic episodes, leading to falls, and bone fractures (109). Alternatively, premixed insulin regimen, eliminating the challenge of mixing insulin, may have a role in elderly patients who have regular eating habits, with similar efficacy as compared with basal bolus therapy (110). Therefore, use of insulin therapy in elderly patients often requires the assistance of a caregiver if patients' abilities are limited.

## Conclusions

Older adults with type 2 diabetes represent a complex and heterogenous age group. Managing diabetes in older age remains an important clinical challenge for all physicians, either primary care providers or specialists. As older diabetic patients present frequently frailty and/or multiple comorbidities, an individualized patient-centered glycemic target is needed in order to achieve a glycemic control avoiding dangerous hypo- and hyperglycemic events. A comprehensive geriatric assessment should be performed at diagnosis of diabetes to better understand cognitive, visual and motor abilities, and coexisting comorbidities. In the choice of anti-hyperglycemic strategies, drugs with proven tolerability, safety, and minimal hypoglycemic risk should be preferred. Anti-diabetes treatment regimens in elderly must be simple, sustainable, and safe to best mirror patients' preferences, wishes, and needs.

## Author Contributions

GB, MIM, KE, and DG conceived the manuscript. ML, GB, and MIM drafted the manuscript. JM, KE, and DG reviewed and edited the manuscript. All authors gave the approval to the final version of the manuscript.

### Conflict of Interest Statement

MIM received a consultancy fee from MSD and has held lectures for Sanofi, Astrazeneca, and Novo Nordisk. JM has held lectures for Astra Zeneca, Boehringer-Ingelheim, Eli Lilly, MSD, Novo Nordisk, Sanofi, and Servier and received research support from Boehringer-Ingelheim, MSD, Novo Nordisk, Sanofi. KE received a consultancy fee from Eli Lilly and has held lectures for Eli Lilly, Sanofi, and Novo Nordisk. DG received a consultancy fee from Eli Lilly and has held lectures for Eli Lilly and Sanofi. The remaining authors declare that the research was conducted in the absence of any commercial or financial relationships that could be construed as a potential conflict of interest.
